# miRNA-Based Potential Biomarkers and New Molecular Insights in Ulcerative Colitis

**DOI:** 10.3389/fphar.2021.707776

**Published:** 2021-07-09

**Authors:** Jing Zhou, Jialing Liu, Yangyang Gao, Liwei Shen, Sheng Li, Simin Chen

**Affiliations:** ^1^ School of Pharmacy, Chengdu University of Traditional Chinese Medicine, Chengdu, China; ^2^ School of Health Preservation and Rehabilitation, Chengdu University of Traditional Chinese Medicine, Chengdu, China; ^3^ Center for Health Policy & Drug Affairs Operation Management, Chengdu University of Traditional Chinese Medicine, Chengdu, China

**Keywords:** ulcerative colitis, microRNA, colitis-associated cancer, inflammatory bowel diseases, gut microbiota

## Abstract

Ulcerative colitis (UC) is a chronic non-specific inflammatory bowel disease, which usually manifests as abdominal pain, diarrhea and hematochezia. The disease often recurs and is difficult to cure. At present, the pathogenesis is not clear, but it is believed that the disease is caused by a complex interaction among immunity, heredity, environment and intestinal microflora disorders. MicroRNA (miRNA) is endogenous single-stranded non-coding RNA of 17–25 nucleotides (nts). They target the 3'Untranslated Region of a target gene and inhibit or degrade the target gene according to the extent of complementary bases. As important gene expression regulators, miRNAs are involved in regulating the expression of most human genes, and play an important role in the pathogenesis of many autoimmune diseases including UC. Studies in recent years have illustrated that abnormal expression of miRNA occurs very early in disease pathogenesis. Moreover, this abnormal expression is highly related to disease activity of UC and colitis-associated cancer, and involves virtually all key UC-related mechanisms, such as immunity and intestinal microbiota dysregulation. Recently, it was discovered that miRNA is highly stable outside the cell in the form of microvesicles, exosomes or apoptotic vesicles, which raises the possibility that miRNA may serve as a novel diagnostic marker for UC. In this review, we summarize the biosynthetic pathway and the function of miRNA, and summarize the usefulness of miRNA for diagnosis, monitoring and prognosis of UC. Then, we described four types of miRNAs involved in regulating the mechanisms of UC occurrence and development: 1) miRNAs are involved in regulating immune cells; 2) affect the intestinal epithelial cells barrier; 3) regulate the homeostasis between gut microbiota and the host; and 4) participate in the formation of tumor in UC. Altogether, we aim to emphasize the close relationship between miRNA and UC as well as to propose that the field has value for developing potential biomarkers as well as therapeutic targets for UC.

## Introduction

Ulcerative colitis (UC) is one of two main inflammatory bowel diseases (IBD), which is a chronic nonspecific inflammation, mainly manifested as long-term inflammation and ulcer of the rectum and colon. The inflammation caused by UC is usually confined to the mucosa and submucosa, and is usually distributed continuously from the rectum to the proximal colon (Rubin et al., 2019). The course of UC is intermittent, with active and inactive periods appearing alternately. Patients in the active stage usually suffer from abdominal pain, diarrhea, bloody stools and weight loss. It is believed that UC is caused by a complex interaction among environmental factors, genetic susceptibility, altered gut microbiota, and immune disorders. UC often recurs, which may lead to intestinal perforation, toxic megacolon, colorectal cancer (CRC) and other complications (Du and Ha, 2020). UC is incurable. At present, treatment is used to achieve clinical symptom relief, mucosal healing and histological relief (Hindryckx et al., 2015; Ng et al., 2017; Ungaro et al., 2017). At present, the diagnosis of UC is through endoscopic examination combined with clinical symptoms, histological analysis, laboratory examination and imaging research (Kobayashi et al., 2020). However, 15–30% of IBD patients can’t be diagnosed with UC or Crohn’s disease (CD), which is called uncertain colitis (Ballard and M'Koma, 2015), which has a negative impact on the future treatment of patients (Soubières and Poullis, 2016). With the increasing incidence and prevalence rate of UC, it has become a global medical burden (Ng et al., 2017). It has become an urgent clinical demand to seek more effective clinical diagnosis, monitoring and treatment of UC.

MicroRNA (miRNA) is a single-stranded non-coding RNA of 17–25 nucleotides (nts), which is a kind of small RNA and highly conserved in different organisms. miRNA participates in regulating various physiological processes, such as cell growth, differentiation, apoptosis and carcinogenesis, including DNA double-strand breaks (DSBs) (Chowdhury et al., 2013; Hu et al., 2017). Unlike small interfering RNAs (siRNA) and Piwi-associated RNAs (piRNA) (Table 1), piRNA is currently found to be associated with the development of human germ cells, but no piRNA has been found to exist in somatic cells (Slack and Chinnaiyan, 2019; Chen et al., 2021). The action patterns of miRNA and siRNA in the human body are similar, but the difference is that miRNA is mostly endogenous RNA while siRNA is mostly exogenous (Senapati et al., 2019). More than 60% of human protein-coding genes contain at least one conserved miRNA binding site (Lam et al., 2015). miRNA has been found to have significant relevance to a variety of diseases, including UC. In this paper, the physiological synthesis and function of miRNAs were reviewed, and the differential expression of miRNAs in UC was summarized. miRNA as a biomarker for UC was discussed. Finally, the action mechanisms of miRNA were discussed, which involved intestinal immune disorder, barrier dysfunction, intestinal flora imbalance and UC canceration.

**TABLE 1:** Comparison of different small RNAs.

Name	Length (nt)	Strand	Occurrence	Complementarity with target mRNA	Function	Evolutionary conservation
miRNA	17–25	Single-stranded	Plants and animals	Partially complementary, so a single miRNA can target hundreds of mRNA types	Regulation of gene translation	Almost conservative in the species
siRNA	21–23	Double-stranded	Plants and lower animals, but mammals do not	Fully complementary, targeting only one mRNA type	Protection against virus intrusion	Hardly conservative in the species
piRNA	24–31	Single-stranded	Metazoans, mostly germline	—	Genome stabilization	Not very conserved

Nt, nucleotide; miRNA, microRNA; siRNA, small interfering RNA; piRNA, Piwi-interacting RNA.

## Overview of miRNAS

miRNA was first discovered and reported in 1993 (Lee et al., 1993), and found in introns, exons or regulatory sequences of the genome, mainly located in the intergenic regions (Galatenko et al., 2018). It exists in various forms, such as single copy, multiple copies or gene clusters (Cantini et al., 2019). More than 2,500 miRNAs have been found in the human genome (Alles et al., 2019). miRNA participates in almost all biological processes in eukaryotic cells (Friedman et al., 2009) and stably exists in almost all body fluids through extracellular vesicles, thus playing a remote regulatory role (Schaefer et al., 2015; Glinge et al., 2017; Yang et al., 2017; Fredsøe et al., 2019; Zamanillo et al., 2019).

**FIGURE 1:**
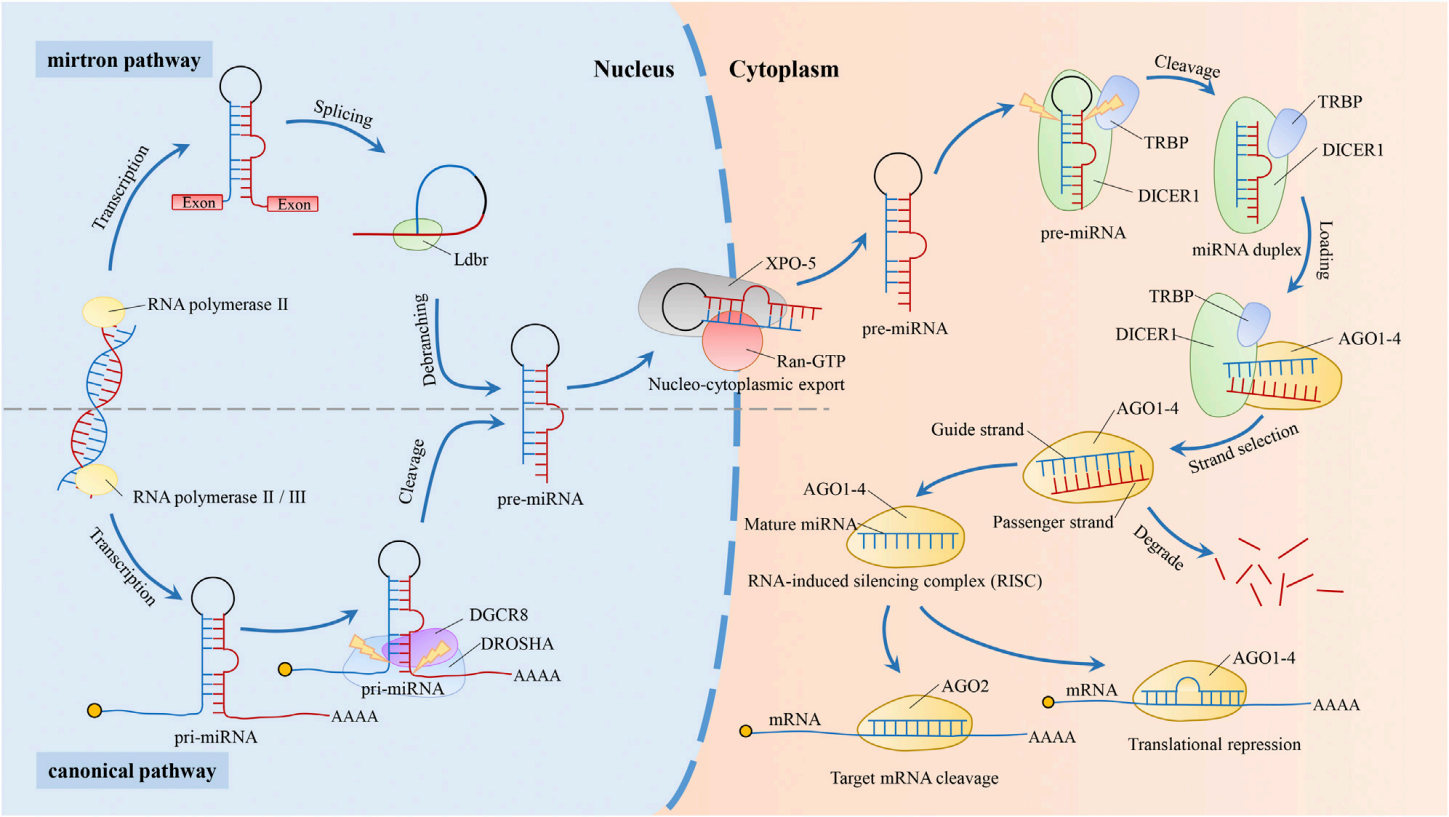
Biogenesis pathway of miRNAs. In the canonical biogenesis pathway of miRNAs. After the miRNA gene is transcribed by RNA polymerase II or III into pri-miRNA, it is sheared by a microprocessor consisting of Drosha and DGCR8 into a pre-miRNA of about 70–100 nucleotides (Rawat et al., 2019). The pre-miRNA is then transported to the cytoplasm by XPO-5 (Yamazawa et al., 2018) and sheared to miRNA duplex by the Dicer-TRBP complex (Fareh et al., 2016; Takahashi et al., 2018). Dicer-TRBP complex binds to AGO1-4 and dissociates after transferring the mature miRNA duplex into AGO1-4 to form RISC. RISC recognizes and binds to target genes by the second to eighth nucleotide counted from 5′-end of the miRNAs (Meijer et al., 2014; Gebert and MacRae, 2019), which is degraded or suppressed the target gene based on the extent of sequence complementarity. Target genes are degraded when completely matched, while inhibited when incompletely matched. During RISC loading, one of the mature miRNAs (the “guide strand”) is retained and forms RISC, while the other is degraded (the “passenger strand”) (Matsuyama and Suzuki, 2019). The non-canonical pathway, mirtron, does not require Drosha. introns are spliced and debranched by the lariat debranching enzyme (Ldbr) to produce pre-miRNA. Similar to the canonical miRNA pathway, this pre-miRNA is transported outside the nucleus via XPO-5. And the mirtron pathway merges with the canonical pathway during this transport stage (Abdelfattah et al., 2014; Stavast and Erkeland, 2019).

In the canonical biogenesis pathway of miRNAs (Figure 1), RNA polymerase II or III transcribes the miRNA gene into the primary transcript pri-miRNA. Then, DGCR8, an RNA-binding protein, binds to Drosha (a type of RNase III) to form the larger Drosha-DGCR8 complex, the microprocessor (Ameres and Zamore, 2013; Kwon et al., 2016). It is a heterotrimeric complex containing one Drosha and two DGCR8 proteins (Nguyen et al., 2015). The Drosha-DGCR8 complex cleaves the pri-miRNA into the intermediate pre-miRNA (Rawat et al., 2019). In this complex, Drosha acts as the catalytic subunit and determines the cleavage site (Ha and Kim, 2014; Nguyen et al., 2015), and DGCR8 stabilizes Drosha through protein-protein interactions, increasing the affinity of Drosha for the substrate and the accuracy of the cleavage site (Han et al., 2009; Ameres and Zamore, 2013; Ha and Kim, 2014; Quick-Cleveland et al., 2014; Nguyen et al., 2015; Rawat et al., 2019). After exportin-5 (XPO-5) and GTP-binding protein (Ran-GTP) form a complex, pre-miRNA is transported to cytoplasm by the complex (Yamazawa et al., 2018). In the cytoplasm, after binding of Dicer and transactivating response RNA binding protein (TRBP), the pre-miRNA is cut into mature double-stranded miRNA of 17–25 nts (Fareh et al., 2016; Takahashi et al., 2018). Then, through loading and strand selection, mature single-stranded miRNA was transferred into Argonaute (AGO) protein by the Dicer-TRBP complex to form an RNA-induced silence complex (RISC). In the past several years, non-canonical biogenesis pathways of miRNAs such as the mirtron (Kim et al., 2016) synthesis pathway continue to be discovered, but most pathways still require Dicer enzymes. The mirtron pathway is the first non-classical pathway discovered. This pathway does not require the Drosha/Dgcr8 complex to generate pre-miRNA, but still requires the transport of XPO-5 and the cleavage of Dicer enzyme (Figure 1) (Abdelfattah et al., 2014; Stavast and Erkeland, 2019).

RISC recognizes and binds to target genes via the seed region (second to eighth nucleotide counted from 5′-end) of miRNA. The canonical pathway of miRNAs mechanism is to recognize target genes by targeting the 3′Untranslated Region (3′-UTR) of mRNA (Kim et al., 2017; Gruszka and Zakrzewska, 2018; Perconti et al., 2019), which is suppressed or degraded based on the extent of sequence complementarity between the target gene and miRNAs (Bartel, 2004). In addition, some studies have reported that a few miRNAs regulate the transcription of target genes through non-canonical pathways. For example, some miRNAs can target the 5′Untranslated Region (5′-UTR) (Kalantari et al., 2017), coding sequences (CDS) (Tang et al., 2017), promoter regions (Matsui et al., 2013), and pri-miRNAs (Tang et al., 2012). miRNAs are widely present in a variety of organisms, including animals, plants and viruses, and most miRNA sequences are highly conserved across organisms and usually have similar functions (Fromm et al., 2015; Bartel, 2018). Of the more than 500 canonical miRNA genes identified in human genome, 296 are conserved among placental mammals (Bartel, 2018). However, for the hundreds of other human miRNAs that are not conserved for mammals, they can not be studied using mouse models. And for miRNAs that are conserved for mice, the mechanisms still need to be validated in human cells (Bartel, 2018).

High-throughput microarray analysis is mainly used to measure changes in miRNA expression profiles, and reverse transcriptase quantitative real-time polymerase chain reaction (RT-qPCR) is used to verify the results of miRNA expression in screening experiments, and to determine the changes of miRNA expression in specific groups. miRNA is abnormally expressed in many diseases including UC, and has been proved to be involved in regulating the immune response of UC and the occurrence and development of colon tumors. Recent studies have also found that miRNA participates in the mutual regulation between host and intestinal flora (Aguilar et al., 2019; Dong et al., 2019; Zhao et al., 2021). These studies of miRNA have provided new insights into the molecular mechanism of UC.

## miRNAs Are Involved in Regulating the Pathogenesis of Ulcerative Colitis

### miRNAs Are Involved in Regulating Immune Cells

**FIGURE 2:**
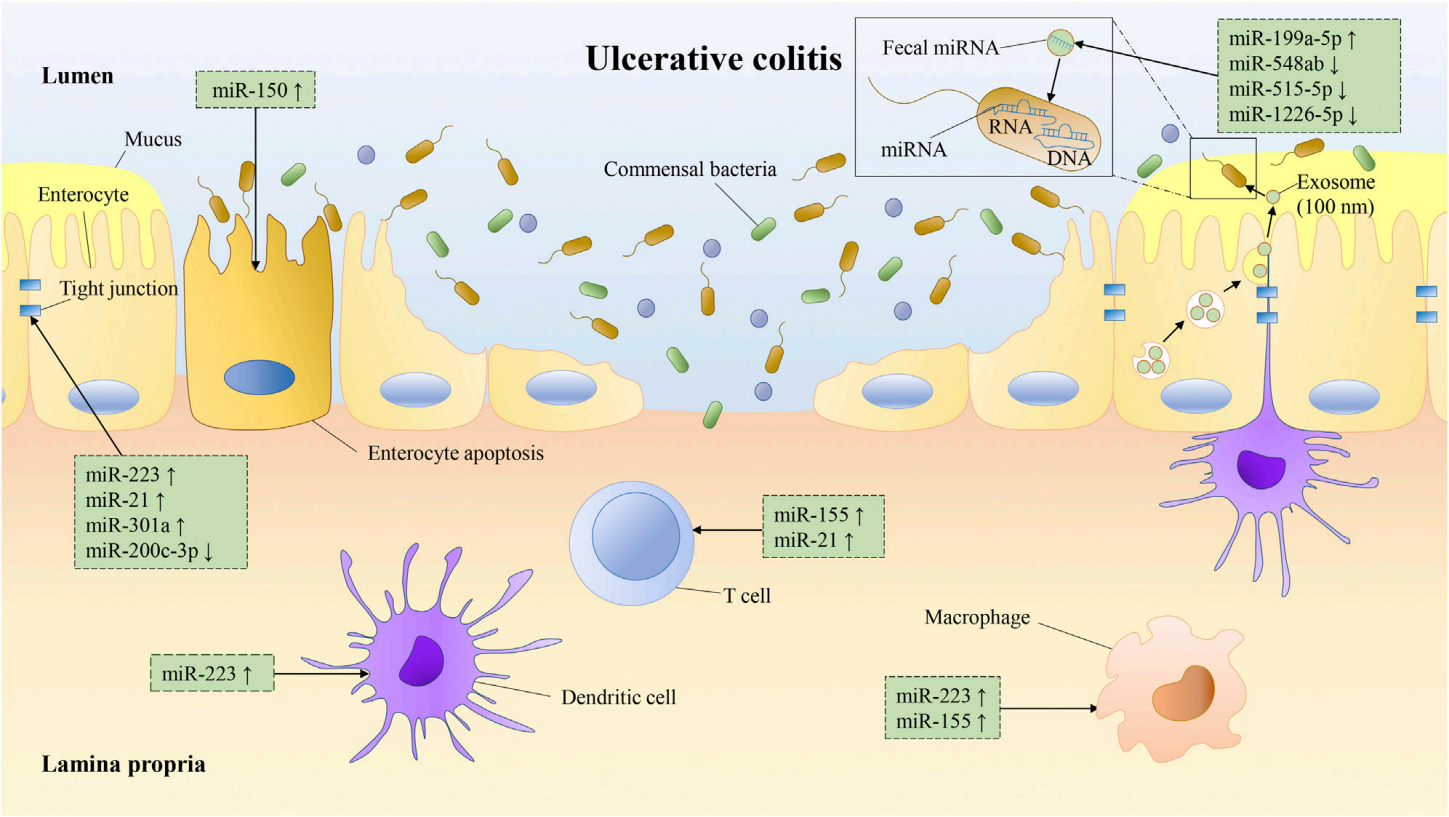
miRNAs are involved in the occurrence and development of UC. miRNAs regulate the generation, differentiation, and function of multiple immune cells (e.g., macrophages, DCs, T-cell) (Murugaiyan et al., 2015; Zhou et al., 2015; Liu et al., 2016a; Wang et al., 2016a; Hou et al., 2017; Neudecker et al., 2017; Xu et al., 2017; Li et al., 2018; Wei et al., 2020). miRNAs also affect the physical barrier of intestinal tract by regulating IEC's tight junctions and apoptosis (Bian et al., 2011; Van der Goten et al., 2014; Zhang et al., 2015; Wang et al., 2016b; He et al., 2017). In addition, miRNAs are secreted by IECs into the intestinal lumen through exosomes and regulate the bacterial growth (Liu et al., 2016b).

Generally, tissues derived from similar anatomical locations in healthy individuals showed no significant differences in the expression of miRNAs (Landgraf et al., 2007; Liang et al., 2007; Martínez et al., 2017). However, the expression of miRNAs appears significantly dysregulated would lead to activation or inhibition of signaling pathways and result in disease (Pallante et al., 2014; Bracken et al., 2016; Hwang et al., 2018). Dysregulation of miRNAs is strongly associated with UC development (Table 2). miRNAs are crucial regulators of intestinal immunity and are involved in the innate and adaptive immune. Thus, they respond to inflammation, and influence the maturation, differentiation, and infiltration of immune cells (Figure 2). miR-141–3p and let-7b/c/f/g-5p are significantly up-regulated in colon intestinal epithelial cells (IECs) of UC mice models and may affect inflammatory cell infiltration by targeting the chemokines CXCL9 or CXCL16 (Lee et al., 2015). miR-223 is significantly up-regulated in patients with active UC and is a potential biomarker closely related to disease activity (see section *miRNAs as Biomarkers for Diagnosis, Monitoring and Prognosis of Ulcerative Colitis*). However, it is reported that miR-223 has an anti-inflammatory role in UC. Myeloid-derived miR-223 reduces IL-1β release via repressing Nlrp3 inflammasome to attenuate experimental colitis (Neudecker et al., 2017). Intestinal macrophages and dendritic cells (DCs) lacking miR-223 exhibit a pro-inflammatory phenotype, and monocytes deficiency of miR-223 promotes an increase in monocyte-derived DCs, resulting in more severe colitis (Zhou et al., 2015). Besides, a high-fat diet promotes the release of exosomes with pro-inflammatory factors (e.g., miR-155) from visceral adipose into the intestine to promote macrophage M1 polarization and aggravate colitis (Wei et al., 2020). Erobic exercise can significantly reduce the expression of miR-155 and miR-146a, and increase miR-126 expression in mouse vascular tissue (Wu et al., 2014). In addition, pro-inflammatory miRNAs, such as miR-23a and miR-155, can be released by activated tissue-infiltrating neutrophils via microparticles (Butin-Israeli et al., 2019). miR-23a and miR-155 can promote the accumulation of DNA double-strand breaks (DSBs) in IECs by downregulating nuclear envelope protein Lamin-B1 and RAD51 (a key homologous recombination regulator), leading to genomic instability and ultimately tumorigenesis (Butin-Israeli et al., 2019).

**TABLE 2:** Differentially expressed miRNAs and its targets in UC.

miRNA name	Target	Sample type	Research object	Expression states	References
miR-26b	DIP1, MDM2, CREBBP, BRCA1	Tissue, blood	Promotes inflammation and CAC by miR-26b/DIP1/DAPK axis	↑	(Benderska et al., 2015)
miR-223	NLRP3	Tissue	Regulate innate immunity in intestinal inflammation	↑	(Neudecker et al., 2017; Macartney-Coxson et al., 2020; Wu et al., 2020)
	CLDN8	Tissue	Regulate IL23/Th17 pathway	↑	(Wang et al., 2016b; Li et al., 2020)
	C/EBPβ	Tissue	Inhibits intestinal macrophages and DCs showing pro-inflammatory phenotype	↑	(Sun et al., 2015; Zhou et al., 2015)
miR-23a	LB1	Tissue	Leads to impaired colon healing and genome instability by promoting DSB accumulation	↑	Butin-Israeli et al. (2019)
miR-155	RAD51	Tissue	Leads to impaired colon healing and genome instability by promoting DSB accumulation	↑	(Gasparini et al., 2014; Butin-Israeli et al., 2019)
	JARID2	Tissue	Induces Th17 cells differentiation	↑	(Xu et al., 2017; Liu et al., 2018b; Zhu et al., 2020b)
	C/EBPβ, SOCS1	Tissue	Regulates the phenotype of macrophages	↑	(Li et al., 2018; Xiao et al., 2019; Zhou et al., 2019)
	IL13RA1	Tissue	Regulates the function of epithelial cells	↑	(Martinez-Nunez et al., 2011; Gwiggner et al., 2018)
miR-301a	BTG1	Tissue	Increases the permeability of IECs and damages the intestinal barrier function	↑	He et al. (2017)
	SNIP1	Tissue, blood	Promotes differentiation of Th17 cells and expression of pro-inflammatory cytokines	↑	He et al. (2016)
miR-200family	Snail	Tissue	Inhibits EMT of colonic mucosa	↓	(Perdigão-Henriques et al., 2016; Zidar et al., 2016)
	Slug	Tissue	Inhibits EMT of colonic mucosa	↓	(Liu et al., 2013; Zidar et al., 2016)
miR-214–3p	STAT6	Tissue	Inhibits IFN-γ expression and intestinal inflammation	↓	Li et al. (2017)
	PDLIM2, PTEN	Tissue	Activates NF-κB pathway and promotes intestinal inflammation	↑	(Polytarchou et al., 2015a; Zhang and Zhang, 2017b; Liu et al., 2019b)
miR-206	A3AR	Tissue	Activates NF-κB pathway and promotes intestinal inflammation	↑	Wu et al. (2017a)
miR-21	PDCD4	Tissue	Activates NF-κB, STAT3 and BCL-2, and promotes the survival of tumor cells	↑	(Ando et al., 2016; Shi et al., 2016; Sun et al., 2020; Hu et al., 2021)
miR-148a-3p	GP130, IKKα, IKKβ, TNFR2	Tissue	Inhibits NF-κB and STAT3 pathways and tumorigenesis	↓	(Zhu et al., 2017; Raso et al., 2019)
miR-148a-5p	IL1R1	Tissue	Inhibits NF-κB and STAT3 pathways and tumorigenesis	↓	Zhu et al. (2017)
miR-133α	AFTPH	Tissue	Promotes intestinal inflammation	↑	(Law et al., 2015; Law et al., 2016)
miR-193a-3p	IL17RD	Tissue	Inhibits carcinogenesis by down-regulating IL17RD	↓	(Pekow et al., 2017; Yu et al., 2019)
miR-31	IL13RA1	Tissue	Regulates the function of epithelial cells	↑	Gwiggner et al. (2018)

↓ indicates inhibition/reduction of miRNA expression in the object described in the corresponding "sample type" item, while ↑ indicates increase/promotion; UC, ulcerative colitis; DAPK, death-associated protein kinase; DIP1, DAPK-interacting protein-1; MDM2, murine double minute-2; CREBBP, cyclic AMP response element-binding protein (CREB)-binding protein; BRCA1, breast cancer genes one; CAC, colitis-associated cancer; NLRP3, NOD-like receptor (NLR) family pyrin domain containing-3; CLDN8, claudin eight; Th17, T helper 17 cell; C/EBPβ, CCAAT/enhancer binding protein beta; DCs, dendritic cells; LB1, lamin B1; DSB, double-strand break; JARID2, jumonji and AT-rich interaction domain containing two; SOCS1, suppressor of cytokine signaling one; IL13RA1, interleukin 13 receptor subunit alpha one; BTG1, B-cell translocation gene-1; IECs, intestinal epithelial cells; SNIP1, Smad nuclear interacting protein one; EMT, epithelial-mesenchymal transition; STAT6, signal transducer and activator of transcription six; IFN-γ, interferon gamma; PDLIM2, PDZ and LIM domain protein two; PTEN, phosphatase and tensin homolog; NF-κB, nuclear factor kappa B; A3AR, adenosine A₃ receptor, also known as ADORA3; PDCD4, programmed cell death protein four; STAT3, signal transducer and activator of transcription three; BCL-2, B-cell lymphoma-2; GP130, glycoprotein 130; IKKα, IκB kinase α; IKKβ, IκB kinase β; TNFR2, tumor necrosis factor receptor two; IL1R1, interleukin one receptor type 1; AFTPH, aftiphilin; IL17RD, interleukin 17 receptor D.

In adaptive immunity, miRNAs regulate the differentiation and function of regulatory T-cell (Treg), T helper 17 cell (Th17), cluster of differentiation eight positive (CD8^+^) T-cell and CD8^+^ B-cell (Xu and Zhang, 2016; Yang et al., 2016). miR-155 could induce Th17 differentiation via targeting Jarid2 (Xu et al., 2017) and is essential for the generation and function of T follicular helper cells (Tfh) (Liu et al., 2016a). Macrophages lacking miR-155 are polarized to the M2 phenotype, attenuating intestinal immune cell proliferation, and inhibiting cluster of differentiation four positive (CD4^+^) T-cell polarization to Th1 and Th17 (Hou et al., 2017; Li et al., 2018). miR-21 also promotes Th2 cell differentiation and is involved in Th2-type inflammation development (Murugaiyan et al., 2015; Wang et al., 2016a).

The above studies indicated that the regulation of miRNAs to suppress inflammation is an effective way to treat UC. It should be noted that since a single miRNA can regulate hundreds of target genes, the effect of anti-inflammatory miRNAs is a combined result presented after acting on various immune cells, rather than on a certain type of immune cells. The therapeutic use of miRNAs mainly includes both inhibition of pro-inflammatory miRNAs and overexpression of anti-inflammatory miRNAs. Viral vectors, lipids, polymers, inorganic and extracellular vesicles are usually used to deliver therapeutic miRNAs (Dasgupta and Chatterjee, 2021). However, due to the wide regulation of miRNA (Lewis et al., 2003; Broughton and Pasquinelli, 2016), how to reduce the regulation unrelated to the therapeutic purpose is the main problem at present.

### miRNAs Affect the IEC’s Barrier

miRNAs are key regulators of the IEC's barrier, regulating the growth and apoptosis of IECs, as well as the tight junctions between IECs (Figure 2). IEC's barrier is the foremost component of the intestinal mucosal barrier, and the intestinal mucosal barrier enables epithelial cells to maintain immune tolerance to more than 10 trillion intestinal microorganisms (Kanneganti, 2017). When the intestinal mucosal barrier is disrupted, intestinal microbes invade the IECs, triggering intestinal inflammation (Jostins et al., 2012). miR-223 is induced by the IL23/Th17 pathway and disrupts the tight junctions between IECs via targeting Claudin-8 (Wang et al., 2016b). miR-21 may also regulate IEC's permeability but by promoting protein kinase B (AKT) phosphorylation and inhibiting PTEN expression through the PTEN/PI3K/AKT signaling pathway. And the knockout of miR-21 reduces intestinal permeability (Zhang et al., 2015). miR-301a is up-regulated in IECs of active IBD patients. It decreases the expression of cadherin-1 and increases cell permeability to disrupt intestinal barrier function and promotes inflammation and tumorigenesis via targeting BTG anti-proliferation factor 1 (BTG1) (He et al., 2017).

In IBD, the miR-200 family may maintain the integrity of IECs and inhibit intestinal fibrosis by inhibiting epithelial-mesenchymal transition (EMT) (Zidar et al., 2016). miR-200b-3p is significantly up-regulated in cancerous epithelial cells and dysplastic tissues of UC patients, which indicates that miR-200b-3p may be involved in the carcinogenesis of UC (Lewis et al., 2017). miR-200c-3p is down-regulated in the mucosa of UC patients in the active stage, the inhibition of its target IL-8 and cadherin-11 (related to the barrier function of IEC) subsequently decreased (Van der Goten et al., 2014). miR-150 is up-regulated in the mucosa of UC patients, and inhibits the expression of target transcription factor c-Myb, resulting in a decrease in the expression of Bcl-2. This pattern suggests that miR-150 may disrupt epithelial barrier function through an apoptotic mechanism (Bian et al., 2011). But miR-150 can also induce apoptosis and inhibit tumor cell migration and invasion, which correlates with CRC patient prognosis (Ma et al., 2012).

In general, miRNA plays a key role in maintaining the function of intestinal tissue barriers. miRNA can enhance the tight junctions between IECs and reduce their permeability, and is related to apoptosis. miRNA not only can be used as potential therapeutic targets for UC, but also has a good applications prospect in maintaining the stability of the intestinal barriers in healthy individuals.

### miRNAs Regulate the Homeostasis Between Gut Microbiota and the Host

Recent studies have reported that miRNAs are involved in the cross-regulation between gut microbiota and host (Figure 2). Intestinal microflora is an important part of the gut microenvironment. UC is usually associated with the proliferation of pathogenic intestinal bacteria (such as *Escherichia coli*, *Salmonella* and *Clostridium difficile*) (Mead et al., 1999).

The expression of some miRNAs in B-cell can be promoted by short-chain fatty acids (a metabolite of intestinal microorganisms). These miRNAs inhibit Aicda and Prdm1 in B-cell, and thus regulate the differentiation of B-cell (Sanchez et al., 2020). Seth and his colleagues found that nitric oxide produced by gut microbes in *Caenorhabditis elegans* can cause S-nitrosylation modification of AGO, a key protein of the miRNA pathway, thereby inhibiting miRNA activity and affecting *C. elegans* development (Seth et al., 2019). Moreover, nitric oxide can modify the S-nitrosylation of AGO proteins in mammals (Seth et al., 2019). In addition, the reduction of intestinal microbial community abundance in specific pathogen-free (SPF) mice mediated by broad-spectrum antibiotics promoted tumor lung metastasis through circRNA/miRNA networks, while tumor metastasis was effectively inhibited by transplantation of fecal bacteria into germ-free (GF) mice using SPF mice feces (Zhu et al., 2020a). *Fusobacterium nucleatum* inhibits the expression of miR-18a* and miR-4802 by activating the TLR4/MYD88 pathway. This reduces the inhibition of miR-18a* and miR-4802 on their target genes ATG7 and ULK1, resulting in activation of the autophagic pathway and alteration of CRC chemotherapy response (Yu et al., 2017).

Conversely, the host can regulate the development of intestinal microbes via miRNAs. Liu and his colleagues found that miRNAs are secreted into the intestinal lumen by IECs via extracellular vesicles and enter the microbes to target mRNAs and regulate microbial development (Liu et al., 2016b). And fecal miRNA from wild-type mice could alleviate dextran sulfate sodium (DSS)-induced colitis in miRNA-deficient mice (Liu et al., 2016b). Subsequently, Liu et al. found that fecal miR-30d targeted *Akkermansia muciniphila* and increased its abundance in the intestine by upregulating lactase expression (Liu et al., 2019a). Ji et al. screened fecal miRNAs that are differentially expressed in IBD, and found that four of these miRNAs (miR-199a-5p, miR-548ab, miR-1226 and miR-515–5p) could target and regulate the proliferation of *Fusobacterium nucleatum*, *Escherichia coli* and *segmental filamentous bacteria* (Ji et al., 2018). In addition, food-derived miRNAs can also regulate the development of gut microbiota. miRNAs from ginger exosomes reduce colitis by promoting the multiplication of *Lactobacillus rhamnosus* and the production of ligands for aryl hydrocarbon receptors (Teng et al., 2018).

miRNAs can also indirectly participate in the regulation of intestinal homeostasis by affecting the expression of intestinal immunoglobulins. Some immunoglobulins promote host-microbiota interactions by binding symbiotic bacteria, which are important for maintaining intestinal homeostasis (Pabst, 2012; Magri et al., 2017; Castro-Dopico et al., 2019). For example, immunoglobulin A (IgA) secretion-deficient mice and humans show increased susceptibility to inflammatory bowel disease, celiac disease and allergy. (Moon et al., 2015). The study showed that miR-221–5p could target polymeric immunoglobulin receptor (pIgR), which is closely related to the sustained supply of secretory IgA, reducing the intestinal secretory IgA level (Bruno et al., 2011; Zeng et al., 2021). Activation-induced cytidine deaminase (AID), a key enzyme of intestinal immune antibody, was targeted by miR-155. Inhibition of miR-155 leads to upregulation of AID and ameliorates the disruption of the intestinal immune barrier caused by IgA and IgM dysfunction (Fairfax et al., 2015; Zhang et al., 2018). Crk-like protein (CRKL) is found to be related to the regulation of B-cell and immunoglobulin G (IgG) levels. miR-29a targets and inhibits CRKL level, and regulates IgG expression through miR-29a/CRKL axis (Shi et al., 2020). In addition, miR-17–92 is reported to be necessary for the production of immunoglobulin G2c (IgG2c) isoforms in B-cell. The IgG generation of miR-17–92-deficient B-cell is significantly reduced in the intestine (Xu et al., 2015; Wu et al., 2018). Currently, we know little about the effects of miRNAs on immunoglobulins in UC, but how miRNAs participate in the interaction between these immunoglobulins and the gut microbiota will be a promising field.

These findings reveal the role of miRNAs in host-microbe interactions, and provide a new method to maintain gut ecological stability.

### miRNAs Participate in the Formation of Tumor in Ulcerative Colitis

**FIGURE 3:**
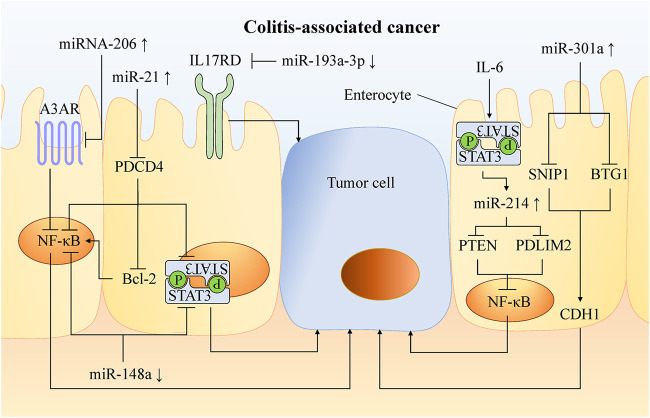
Mechanism of miRNA involved in carcinogenesis of UC. In UC, chronic activation of carcinogenic pathways such as NF-κB and STAT3 leads to a higher risk of CAC. A variety of miRNAs can promote or inhibit the occurrence of cancer by targeting molecules in these pathways. Increasing the abundance of anti-oncogenic miRNAs and decreasing the abundance of oncogenic miRNAs can inhibit tumor formation and progression in experimental colitis. This provides a new therapeutic target for the preventing and treatng CAC.

Patients with UC for more than 8 years have an increased risk of colitis-associated cancer (CAC) (Chen et al., 2016), and it is an urgent clinical need to prevent UC from developing into CRC. At present, it is believed that the sustained activation of carcinogenic signaling pathways, such as NF-κB, STAT3 (Li et al., 2017), and PI3K (Zhang et al., 2015), the release of pro-inflammatory mediators and the increase of local levels of reactive oxygen species and nitrogen substances, contribute to the development of CAC. Now it has been found that some miRNAs can regulate the carcinogenesis of UC (Figure 3). miR-301a promotes Th17 differentiation and decreases cadherin-1 expression to disrupt intestinal barrier function by targeting SNIP1 and BTG1. And knockout of miR-301a reduced inflammation and inhibited tumor occurrence (He et al., 2016; He et al., 2017).

NF-κB is an essential pathway that promotes tissues with inflammation to transform into cancer. Through molecules targeting this pathway, a variety of miRNAs can be involved in cancer formation. For example, miR-206 is up-regulated in the mucosa of UC patients, and promotes intestinal inflammation by targeting the adenosine A3 receptor (A3AR) to reduce the inhibition of NF-κB pathway (Wu et al., 2017a). miR-214 is highly expressed in the colonic tissues of active UC and CAC patients by STAT3 pathway. It activates the NF-κB pathway by targeting and inhibiting PDLIM2 and PTEN expression. miR-214 inhibitors significantly inhibit (>90%) NF-κB phosphorylation, thus reducing the severity of colitis and the number and size of tumors in mice (Polytarchou et al., 2015a).

miR-21 is significantly up-regulated in tissues of UC (Yan et al., 2020), CRC (Wu et al., 2017b) and CAC, activates the NF-κB, STAT3, and Bcl-2 by targeting PDCD4 (Figure 3). Then, it reduced the apoptosis of mouse tumor cells (Shi et al., 2016). Blocking miR-21 reduced pro-inflammatory and pro-cancer factors, and then decreased the number and size of colon tumors in mice (Shi et al., 2016).

miR-148a is down-regulated in the colon tissues of UC and CRC patients, and reduces the activation of NF-κB and STAT3 in macrophages of colon tissues by directly targeting upstream regulators of NF-κB and STAT3 pathway (including GP130, IKKα, IKKβ, IL1R1 and TNFR2). miR-148a-deficient mice are more susceptible to UC and CAC (Zhu et al., 2017).

miR-155 is up-regulated in a variety of malignant tumors including CRC, which promotes proliferation and metastasis of colon tumor cells, and is related to tumor location, tumor grade, metastasis and TNM stage (Qu et al., 2015). In CRC, miR-155 targets protein tyrosine phosphatase receptor J-type (PTPRJ) mRNA, inhibits the anti-proliferation effects of PTPRJ, promotes the proliferation and migration of tumor cells, and activates the AKT pathway (Zhang et al., 2017a). Al-Haidari et al. found that miR-155–5p upregulates the human antigen R (HuR) by targeting the AU-rich elements (AREs) in the 3′-UTR region of HuR mRNA, thus promoting the metastasis of colon cancer cells. The migration of colon cancer cells can be inhibited by blocking the binding of miR-155–5p and ARE in HuR (Al-Haidari et al., 2018).

These studies prove that miRNA is related to the degree of inflammation and canceration in UC. The pro-inflammatory miRNAs can promote the occurrence and development of cancer, whereas anti-inflammatory miRNAs can also resist cancer. miRNA is involved in the transformation of inflammation to cancer in epigenetics, and thus provides a potential therapeutic target for UC and CRC.

## miRNAs Are Specifically Expressed in Ulcerative Colitis

### miRNAs as Biomarkers for Diagnosis, Monitoring and Prognosis of Ulcerative Colitis

**FIGURE 4:**
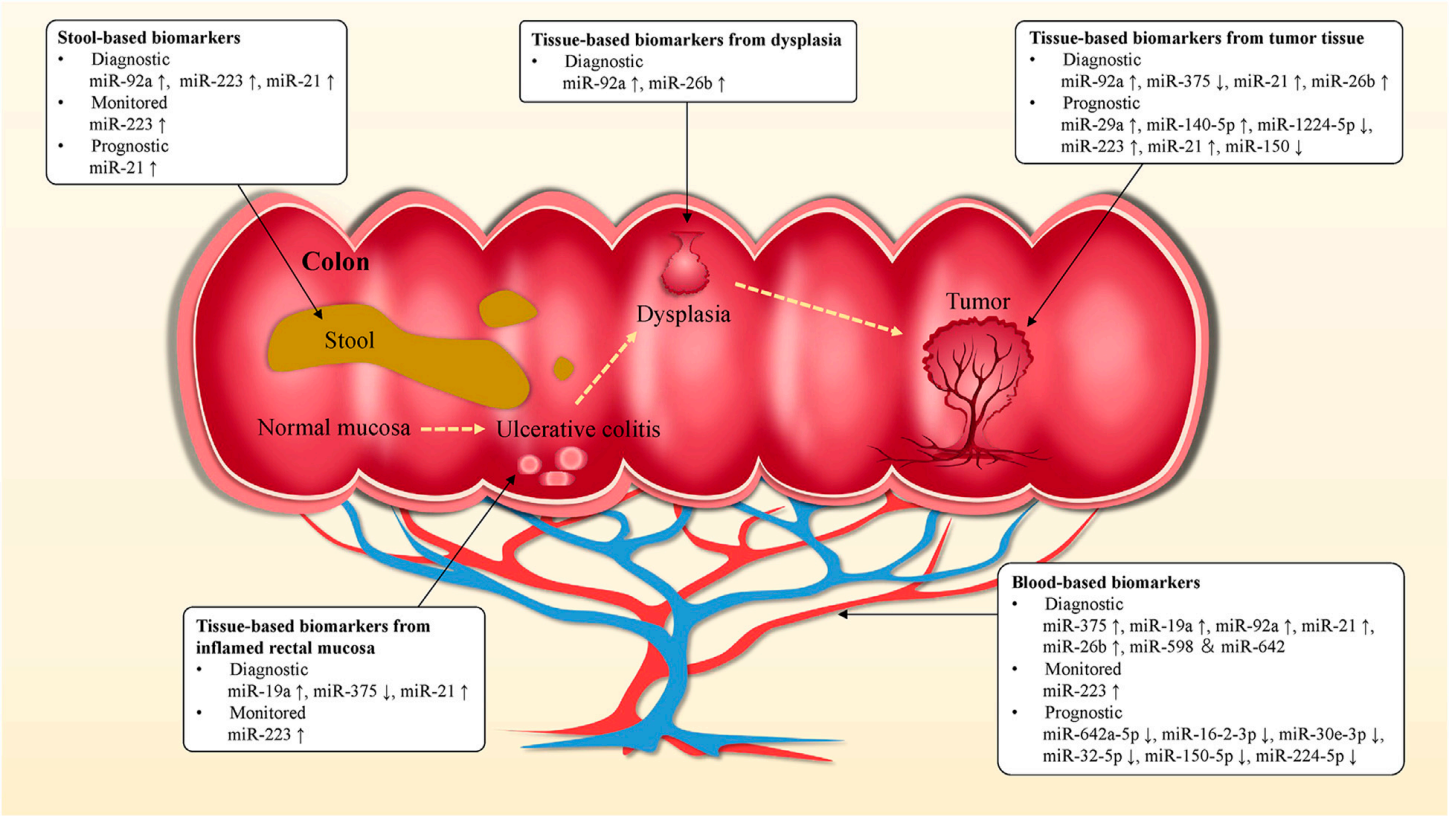
miRNA biomarkers in UC. Blood, stool and tissue-based miRNA biomarkers might be used for the diagnosis, monitoring, prognosis of UC and the prediction of early stage CAC, as well as to guide clinical treatment and medication strategies. miRNA biomarkers could be of value in improving survival and cure rates in UC and CAC patients.

The early diagnosis of UC can provide more accurate drug treatment and prevent complications. At present, colonoscopy is the gold standard in the diagnosis of UC, supplemented by blood tests and biomarker analysis. However, colonoscopy is invasive, which brings considerable physical and economic burden to patients, accompanied by serious complications such as intestinal perforation and death (Hagel et al., 2012; Carmona et al., 2013; Adler et al., 2014; Borgaonkar et al., 2016; Niikura et al., 2016; Vatandoost et al., 2016). We summarized the promising miRNA biomarkers in UC (Table 3) and discussed the prospect of miRNA biomarkers in the diagnosis, surveillance, and prognosis of UC, as well as in the screening for CAC (Figure 4).

**TABLE 3:** miRNAs levels of UC patients in different studies.

miRNA name	Sample type	Disease	Control	Sample size	Expression states	Sensitivity (%)	Specificity (%)	References
miR-223	Feces	aIBD	iIBD	aIBD/iIBD: 30/15	↑	80	93	Schönauen et al. (2018)
	Feces	aUC	HC	aUC/HC: 10/15	↑	90	87	Schönauen et al. (2018)
	Serum	aUC	iUC	aUC/iUC: 24/22	↑	79	72	Polytarchou et al. (2015b)
miR-21	Plasma	aUC	IBS/HC	aUC/IBS/HC: 37/30/30	↑	88	92	Ahmed Hassan et al. (2020)
miR-92a	Plasma	aUC	IBS/HC	aUC/IBS/HC: 37/30/30	↑	88	100	Ahmed Hassan et al. (2020)
miR-4454	Serum	aUC	iUC	aUC/iUC: 24/22	↑	70	68	Polytarchou et al. (2015b)
Panel of (miR-598/miR-642)	Plasma	UC	CD	UC/CD: 21/12	↑/↑	72	86	Netz et al. (2017)
miR-23a-3p	Serum	aUC	iUC	aUC/iUC: 24/22	↑	79	68	Polytarchou et al. (2015b)
miR-320e	Serum	aUC	iUC	aUC/iUC: 24/22	↑	67	67	Polytarchou et al. (2015b)
miR-16-2-3p	Serum	GR	GS	GR/GS: 37/39	↓	74	97	Luo et al. (2018)
miR-30e-3p	Serum	GR	GS	GR/GS: 37/39	↓	85	89	Luo et al. (2018)
miR-32–5p	Serum	GR	GS	GR/GS: 37/39	↓	97	60	Luo et al. (2018)
miR-642a-5p	Serum	GR	GS	GR/GS: 37/39	↓	92	73	Luo et al. (2018)
miR-150–5p	Serum	GR	GS	GR/GS: 37/39	↓	67	97	Luo et al. (2018)
miR-224–5p	Serum	GR	GS	GR/GS: 37/39	↓	90	97	Luo et al. (2018)

↓ indicates inhibition/reduction of miRNA expression in the condition described in the corresponding "disease" and "sample type" item, while ↑ indicates increase/promotion; UC, ulcerative colitis; aIBD, active inflammatory bowel diseases; iIBD, inactive inflammatory bowel diseases; aUC, active ulcerative colitis; HC, healthy control; iUC, inactive ulcerative colitis; IBS, irritable bowel syndrome; CD, Crohn's disease; GR, glucocorticoid-resistant; GS, glucocorticoid-sensitive.

Tissue, blood and stool samples were mainly used in the study of miRNAs in UC. For example, plasma miR-21 and miR-92a are up-regulated in UC and could distinguish active UC (*n* = 37) from non-IBD (30 healthy individuals and 30 patients with irritable bowel syndrome (IBS)), with the specificity of 92 and 100%, respectively, and sensitivity of 88% (Ahmed Hassan et al., 2020). Among them, miR-21 level is positively correlated with histologically assessed disease severity, which is closely related to the development of inflammation. And miR-21 increases IEC permeability and promotes Th2-type inflammation (Murugaiyan et al., 2015; Wang et al., 2016a). Recent studies have shown that circulating miR-375 is significantly up-regulated in UC patients compared to healthy controls and CD patients, which is a potential biomarker for the diagnosis of UC (Schaefer et al., 2015). Further, it may be a potential early biomarker for CAC. Patel et al. reported the analysis of plasma miR-375 in UC patients (*n* = 37), UC patients with dysplasia (*n* = 2) and CAC patients (*n* = 6) (Patel et al., 2015). They found that plasma miR-375 in CAC patients was significantly up-regulated. Notably, miR-375 has low expression in colon tissues of UC and CRC patients, and participates in the regulation of epidermal growth factor receptor signal pathway by targeting the connective tissue growth factor (Alam et al., 2017). miR-375 affects the growth and invasion of colon cells, and overexpression of it can enhance apoptosis and necrosis (Alam et al., 2017; Garrido-Mesa et al., 2018).

High recurrence rate is still one of the main characteristics of UC, and the evaluation of patients' clinical symptoms has a great impact on treatment decision-making and nursing management. At present, the indicators used to monitor the activity of UC all have their own limitations, which makes the nursing care of patients with UC complicated (Tsilidis et al., 2008; Henderson et al., 2015; Mosli et al., 2015; D'Angelo et al., 2017; Fabian et al., 2019). Recently, miRNA in circulation and feces has become a potential non-invasive biomarker for monitoring disease activity of IBD patients, and it is significantly related to endoscopic activity. For example, miRNA-146a is down-regulated in UC patients, which is negatively correlated with Sutherland Disease Activity Index (DAI) score, clinical activity index and endoscopy index (Feng et al., 2020). The research showed that miRNA-146a could relieve colitis and CRC by targeting TRAF6, PTGES2, and TLR4/MyD88/NF-κB signaling pathways (Wang et al., 2019; Garo et al., 2021). Compared to healthy controls, the expression of serum miR-146b-5p in UC is up-regulated by 2.72 fold and significantly correlates with disease activity (Chen et al., 2019). This may be related to miR-146b promoting colonic mucosal repair. miR-146b can strongly inhibit the activation of M1 macrophages by suppressing the Toll-like receptor 4 (TLR4) signaling pathway, thus inhibiting the induction of pro-inflammatory cytokines. (Deng et al., 2019). In addition, miR-223 increased significantly in feces, tissue and serum samples of UC patients with the active stage (Wang et al., 2016c; Schönauen et al., 2018; Mohammadi et al., 2019; Verdier et al., 2020). And it is significantly up-regulated compared with non-inflammatory areas in colonoscopy biopsy of UC patients (Valmiki et al., 2017). Previous studies have shown that miR-223 helps to alleviate colonic inflammation in UC patients (Zhou et al., 2015; Neudecker et al., 2017). However, the studies of biomarkers show that the expression level of miR-223 is positively correlated with disease activity in UC patients. The sensitivity and specificity of fecal-derived miR-223 in distinguishing active IBD patients (fecal calprotectin >500 μg/g and active disease in colonoscopy, *n* = 30) from remission IBD patients (fecal calprotectin <100 μg/g and remission in colonoscopy, *n* = 15) was 80 and 93%, respectively (Schönauen et al., 2018). Wang et al. (2016c) analyzed serum samples from 50 UC patients, 50 CD patients and 50 healthy controls, and found that serum miR-223 level in UC patients was positively correlated with Mayo Endoscopic Score (MES), erythrocyte sedimentation rate (ESR), high-sensitivity C-reactive protein (hs-CRP) and Ulcerative Colitis Endoscopic Index of Severity (UCEIS). And miR-223 (*r* = 0.481) has a higher Spearman *r* value than ESR (*r* = 0.334) when correlated with UCEIS. Likewise, Polytarchou et al. (Polytarchou et al., 2015b) analyzed serum miR-223 levels in 21 healthy controls and 46 UC patients (22 in remission, 10 with moderately active disease and 14 with severe active disease) and found that miR-223 was positively correlated with MES and C-reactive protein (CRP). The Spearman *r* value of miR-223 (*r* = 0.44) is higher than that of CRP (*r* = 0.30) when it is related to MES. But miR-223 is highly expressed in platelets, and improper treatment during serum separation can lead to the introduction of miR-223 into platelets, thus these may affect the experimental results (De Guire et al., 2013).

Currently, some studies have reported the use of miRNA biomarkers for predicting response to treatment, disease recurrence, and the severity of disease over time or something else in UC. Although this area has not been adequately studied, it does represent a valuable direction. Malham et al. found that the expression level of miR-21 was significantly lower in pediatric UC patients than in adult UC patients, and was greater in male patients than in female patients in the adult group (Malham et al., 2021). Batra et al. screened for biomarkers associated with clinical response to anti-TNF-α therapy and glucocorticoid (GC) therapy in pediatric IBD patients and identified five serum miRNAs (miR-146a, miR-146b, miR-320a, miR-126, and let-7c), which expression correlated not only with disease mucosal biopsy but also with treatment response and are potential non-invasive biomarkers for clinical monitoring (Batra et al., 2020). It was found that miR-206 levels were elevated in UC and associated with inhibition of anti-inflammatory A3 adenosine receptor (A3AR) expression, and miR-206 expression was reduced in mesalazine-treated colon cells as well as in colon tissue from patients treated with mesalazine (Minacapelli et al., 2019). A prospective study was conducted by Kalla et al., which found that miR-3615 and miR-4792 in T-cell in the blood contributed to the prognosis of UC patients. When patients meet at least three criteria of the four biomarkers [relative miR-3615 expression <0.95, miR-4792 >2.26, albumin <39 g/dl and extensive colitis (logrank *p* = 6.93 × 10^7^)], there was a 90% probability that the patient would require treatment escalation within one year (Kalla et al., 2020). Luo et al. reported that six miRNAs were significantly down-regulated in the serum of GCs-resistant UC patients and these could be used to identify GCs-resistant UC patients. In particular, miR-224–5p is a strong predictor with an area under the curve (AUC) = 0.99, specificity = 97.30%, and sensitivity = 89.70% (Luo et al., 2018). Similarly, Morilla et al. used a panel of miRNA biomarkers (has-miR-3934, hsa-miR-100, hsa-miR-718, hsa-miR-193b, hsa-miR-3150a-5p, hsa-miR-1260b, hsa-miR-938, has-miR-518b, and hsa-miR-1468) to distinguish steroid-resistant acute severe ulcerative colitis (ASUC) patients with high accuracy (AUC = 0.87) (Morilla et al., 2019). Jabandziev et al. analyzed miRNA expression in colonic tissue of 60 pediatric UC patients and found four panels of miRNAs, which were used to distinguish pediatric UC patients from healthy controls (let-7i-5p, miR-223–3p and miR-4284, AUC = 0.99), to detect severity in pediatric UC patients (miR-375–3p, miR-146a-5p, miR-223–3p and miR-200b-3p, ACU = 0.73), to detect disease relapse within one year of treatment (miR-21–5p, miR-192–5p and miR-194–5p, AUC = 0.73) and to diagnose UC patients with primary sclerosing cholangitis (miR-142–3p, miR-146a-5p, miR-223–3p, let-7i-5p, miR-192–5p and miR-194–5p, AUC = 0.858) (Jabandziev et al., 2021).

The above studies demonstrate that miRNAs are promising potential biomarkers of UC. The quantitative detection of miRNA has high sensitivity and accuracy, and is related to the activity of UC. However, at present, most studies only detect the changing trend of miRNA expression, but do not quantitatively analyze miRNA. In future research, it is necessary to standardize the sample detection scheme, and detect the abundance of miRNA in UC, so as to determine the accurate expression range of potential miRNA biomarkers. In addition, at present, there are few studies on how the drugs commonly used in UC, the length of duration of the disease, the age of patients, and other factors affect miRNA expression, but this is indeed a promising field.

### miRNAs as Biomarkers for the Diagnosis of Colitis-Associated Cancer

Investigations have shown that patients with long-term UC have an increased risk of tumor complications (Chen et al., 2016), while patients with CAC have a poor prognosis and higher mortality than patients with sporadic CRC (Dugum et al., 2017). In addition, the five-year survival rate for diagnosis at early stages of CRC is 90%, compared to 13% for stage IV (Bray et al., 2018). As important post-transcriptional regulators, some miRNAs are abnormally expressed in the carcinogenesis of UC (Table 4), which can be used for the diagnosis of early canceration.

**TABLE 4:** miRNA levels of CAC or CRC patients in different studies.

miRNA name	Sample type	Disease	Control	Sample size	Expression states	Sensitivity (%)	Specificity (%)	References
miR-375	Plasma	CAC	UC/UCD	UC/UCD/CAC: 37/2/6	↑			Patel et al. (2015)
miR-21	Plasma	CRC	UC	UC/CRC: 37/33	↑	94	100	Ahmed Hassan et al. (2020)
miR-92a	Plasma	CRC	UC	UC/CRC: 37/33	↑	84	100	Ahmed Hassan et al. (2020)
	Feces	CRC	HC	CRC/HC: 29/29	↑	90	52	Choi et al. (2019)
Panel of (miR-223/miR-92a)	Plasma, feces	CRC	HC	CRC/HC: 62/40	↑/↑	97	75	Chang et al. (2016)
miR-200b-3p	Tissue	UCD	UC	UCD/UC: 10/7	↑			Lewis et al. (2017)
miR-144*	Feces	CRC	HC	CRC/HC: 29/29	↑	79	67	Choi et al. (2019)

↓ indicates inhibition/reduction of miRNA expression in the condition described in the corresponding "disease" and "sample type" item, while ↑ indicates increase/promotion; CAC, colitis-associated cancer; CRC, colorectal cancer; UC, ulcerative colitis; UCD, ulcerative colitis-associated dysplasia; HC, healthy control.

miR-21, one of the most studied miRNA in UC, is up-regulated in blood (Ahmed Hassan et al., 2020), feces (Schönauen et al., 2018), and colonic tissues (Yan et al., 2020) of UC patients and promotes the development of intestinal inflammation (Murugaiyan et al., 2015; Wang et al., 2016a). miR-21 can activate NF-κB, STAT3 and bcl-2 signaling pathways by targeting PDCD4, thus reducing apoptosis in tumor cells (Figure 3). Compared to the expression in UC patients, miR-21 further increased in CRC (Wu et al., 2017b; Choi et al., 2019; Ahmed Hassan et al., 2020) where it regulated the physiological function of tumor cells (Wu et al., 2017b). Therefore, detection of miR-21 level might be an effective method to assess the risk of carcinogenesis in patients with UC. A meta-analysis confirmed that miR-21 is the most reliable potential fecal-based miRNA biomarker of CRC, with an AUC of 0.843, sensitivity of 59.3% and specificity of 85.6% (Yau et al., 2019). Ahmed Hassan et al. (Ahmed Hassan et al., 2020) measured plasma miR-21 expression in 37 patients with active UC, 33 with CRC, and 30 with IBS as well as 30 healthy controls. The results showed that the sensitivity and specificity of distinguishing UC from IBS and healthy controls were 87.5 and 91.7% (AUC = 0.844). The sensitivity and specificity of miR-21 in distinguishing CRC from UC were 93.5 and 100%, respectively. In addition, miR-26b is up-regulated in the progression of UC to CAC and down-regulated in sporadic CRC, thus distinguishing CAC from UC and sporadic CRC (Benderska et al., 2015). Furthermore, research has shown that miR-26b inhibits tumor cell proliferation and invasion, and induces apoptosis of tumor cells. Compared with paired primary CRC tissues, the expression level of miR-26b was almost 4-fold higher in lung metastases, which may be associated with lung metastasis (Cristóbal et al., 2015).

Detection of miRNA methylation, rather than expression, is another accurate diagnostic method for CAC. Methylation changes the stability and specific recognition of miRNA (Konno et al., 2019), and the methylation level is related to the age and course of UC patients (Toiyama et al., 2017). miRNA methylation in UC patients with dysplasia or CRC increased significantly (Toiyama et al., 2017). Toiyama et al. accurately diagnosed UC patients with dysplasia and CRC by detecting a panel of methylated miRNA (evaluation cohort AUC = 0.81; validation cohort AUC = 0.78) (Toiyama et al., 2017).

Compared with the traditional blood-based biomarkers (CEA or CA 19–9), miRNA expression has been found to significantly different in every stage of CRC and has a higher diagnostic value for early diagnosis, disease monitoring and prognosis of CRC (Inoue et al., 2017; Liu et al., 2018a; El-Daly et al., 2019; Baassiri et al., 2020).

## Discussion

Currently, miRNA, as a biomarker of UC, has the advantage of early diagnosis and better correlation with disease activity (Wang et al., 2016c; Schönauen et al., 2018). However, because miRNA disorders involve many diseases, how to ensure miRNA biomarkers in UC are not interfered by other diseases or therapies is a challenge in the future (Kalla et al., 2015). In future studies of miRNA in UC, it is necessary to study the role of miRNA in specific tissues and cell types (Whiteoak et al., 2015). In addition, some immunoglobulins, such as sIgA and IgG, are not only important molecules of the intestinal mucosal immune barrier, but also the main effective antibodies of the humoral immune response, which play an important role in the pathological mechanism of UC. Castro-Dopico et al. reported that IgG drives intestinal inflammation and Th17-related immunity in the mucosal immune hyperactivation caused by genetic variants of UC (Castro-Dopico et al., 2019). sIgA has been reported to bind intestinal microbes and regulate the composition of the intestinal microbiota (Okai et al., 2016). As post-transcriptional regulators, the interaction between miRNAs and immunoglobulins in intestinal immunity is also a field of interest in the future.

At present, miRNA biomarkers in UC are mainly studied for clinical diagnosis and monitoring, however, miRNA biomarkers seem to have other values. Due to the broad regulatory effects of miRNA, it is associated with almost all clinical reactions, such as in predicting disease recurrence (Jabandziev et al., 2021), therapeutic escalation (Kalla et al., 2020), and response to drug therapy (Batra et al., 2020). Although this aspect is currently under-researched, they do represent a valuable direction of research. The current studies show that miR-21 seems to be the most promising potential miRNA biomarker for UC. miR-21 is up-regulated in blood, feces, and colonic tissues of UC patients and further increased in CRC (Wu et al., 2017b; Schönauen et al., 2018; Choi et al., 2019; Ahmed Hassan et al., 2020; Yan et al., 2020), which allows it to differentiate UC from non-IBD (healthy individuals and IBS) and also for the diagnosis of CAC. And the expression of miR-21 was correlated with the age and gender of UC patients (Malham et al., 2021). Moreover, fecal-derived miR-21 has no significant changes in CD, which increases the possibility that miR-21 may be used to distinguish UC from CD (Zhou et al., 2021). miR-223 has a higher correlation with disease activity than ESR and CRP, and is the most promising potential biomarker for monitoring disease activity in UC (Polytarchou et al., 2015b; Wang et al., 2016c). In addition, since most miRNA biomarkers are tested in small sample sizes, they must be verified in large-scale longitudinal studies before being applied to clinical practice. On the other hand, there is no uniform quantitative standard for the detection of miRNA, which is one of the reasons limiting the development of miRNA biomarkers. Especially for complex samples, such as feces, there is a need for more effective and standardized measurement method (Niu et al., 2019). Measuring exosomal-derived miRNAs in blood may reduce the interference of other components, but the technology and cost of isolating exosomes are also challenging issues (Vasconcelos et al., 2019). Detection of fecal exosomal-derived miRNAs may be a better approach. Fecal exosome-derived miRNAs have been proved to be secreted predominantly by IECs and Hopx-positive cells (Liu et al., 2016b), thus allowing a more accurate assessment of disease activity or mucosal healing in UC.

miRNA is regarded as a potential target for the treatment of UC. But one miRNA can regulate hundreds of proteins, then how to reduce the influence on non-target proteins is the main problem of miRNA-based treatment. At present, this problem is mainly solved by improving drug carriers or finding more specific targets. It was found that plant-derived exosomes can be preferentially absorbed by specific intestinal microorganisms (Teng et al., 2018). Gut microbiota dysbiosis contributes to the pathogenesis of UC, and targeted inhibition of specific pathogenic bacteria using exosomal-derived miRNAs may be a novel approach to reduce off-target effects. Of course, local medication may be the most effective method to reduce systemic exposure. For example, Alicaforsen is currently in clinical trial stage, which is an oligonucleotide therapeutic drug, targeting mRNA and then inhibits the generation of protein. A phase IV clinical trial showed that it is equivalent to mesalazine enema (Miner et al., 2004; Miner et al., 2006a; Miner et al., 2006b). On the whole, although there are still some problems to be solved, miRNA is a promising target in the treatment and diagnosis of UC.
